# Esophageal Rupture as a Primary Manifestation in Eosinophilic Esophagitis

**DOI:** 10.1155/2014/673189

**Published:** 2014-05-11

**Authors:** Natalia Vernon, Divyanshu Mohananey, Ehsan Ghetmiri, Gisoo Ghaffari

**Affiliations:** Penn State Hershey Medical Center, 500 University Drive, MC H041, Hershey, PA 17033, USA

## Abstract

Eosinophilic esophagitis (EoE) is a chronic inflammatory process characterized by symptoms of esophageal dysfunction and, histologically, by eosinophilic infiltration of the esophagus. In adults, it commonly presents with dysphagia, food impaction, and chest or abdominal pain. Chronic inflammation can lead to diffuse narrowing of the esophageal lumen which may cause food impaction. Endoscopic procedures to relieve food impaction may lead to complications such as esophageal perforation due to the friability of the esophageal mucosa. Spontaneous transmural esophageal rupture, also known as Boerhaave's syndrome, as a primary manifestation of EoE is rare. In this paper, we present two adult patients who presented with esophageal perforation as the initial manifestation of EoE. This rare complication of EoE has been documented in 13 other reports (11 adults, 2 children) and only 1 of the patients had been previously diagnosed with EoE. A history of dysphagia was present in 1 of our patients and in the majority of previously documented patients. Esophageal perforation is a potentially severe complication of EoE. Patients with a history of dysphagia and patients with spontaneous esophageal perforation should warrant an evaluation for EoE.

## 1. Introduction

Eosinophilic esophagitis (EoE) is a chronic inflammatory process and a clinicopathological diagnosis. EoE is clinically characterized by symptoms of esophageal dysfunction. Histopathologically, eosinophilic infiltration of the esophagus is the characteristic finding [1]. The diagnosis is made in symptomatic patients after a biopsy that confirms at least 15 eosinophils per high power field (HPF) in the absence of other causes [1–3]. EoE can present in children as well as in adults. Presenting symptoms include dysphagia, odynophagia, food impaction, heartburn, and chest or abdominal pain [3]. The main complication results from chronic inflammation leading to a segmental or diffuse narrowing of esophageal lumen. The narrowing may cause food impaction which may induce vomiting and/or require surgical interventions. Esophageal dilatation through endoscopic procedures to resolve food impaction may lead to partial or complete tear of the already friable esophagus [4]. Partial or complete tear of the esophagus as a primary manifestation of EoE is rare. This paper presents two adult cases of spontaneous esophageal rupture as a primary manifestation of EoE along with a literature review of this rare complication.

## 2. Review of Two Cases

### 2.1. Case 1

A 48-year-old man presented to the emergency department (ED) with progressive chest pain which intensified with inspiration. Earlier that day for the first time, he had had an episode of impaction of a fish oil tablet which was resolved by inducing vomiting. This is when the chest pain started and progressively increased. In the ED, he was initially worked up for a cardiac event and pulmonary embolus, both of which were negative. A computed tomographic (CT) scan of his chest revealed thickening of the esophageal wall as well as evidence of air/gas that appeared to dissect a plane of the esophagus. The CT also revealed extraluminal air suggestive of esophageal perforation. An upper endoscopy with stent placement was performed. The esophageal biopsy taken at this time showed inflammation with increased eosinophils in both the upper and lower esophagus parts. Histological sections of the esophagus showed basal cell hyperplasia, interstitial edema, and intraepithelial eosinophils up to 30 eosinophils per high power field (HPF) in the proximal and distal esophagus. The patient was discharged on a liquid diet, antibiotics, and a proton pump inhibitor. The stent was left in situ for 17 days and then was removed without difficulty. A repeat endoscopy three months later showed basal cell thickening, intercellular edema, and up to 20 eosinophils/HPF in the proximal and distal esophagus. This endoscopy also revealed stricture in the total esophagus and a ringed esophagus consistent with EoE. He was then referred to the allergy/immunology clinic for further evaluation. The clinical picture along with pathological findings was consistent with a diagnosis of EoE. Following the diagnosis of EoE, the patient began treatment with topical fluticasone propionate with complete symptom resolution. Repeat endoscopy on topical steroid for several months showed up to 3 eosinophils/HPF and mild inflammation and the patient had resolution of his symptoms.

The patient had a long standing history of dysphagia for the past 20–25 years predominantly with solid foods. He usually needed to chew his food for extended duration to swallow it with ease. He also reported intermittent symptoms of gastroesophageal reflux in the past which were neither evaluated nor treated. He has had no history of food allergies, asthma, or allergic rhinitis. His past medical history was significant only for hypercholesterolemia. There was no family history of atopic diseases. He consumes alcohol occasionally and does not smoke. A skin prick test done as part of the allergy workup was mildly positive to shellfish.

### 2.2. Case 2

A 63-year-old woman presented to the ED with acute onset of chest pain, nausea, and vomiting after eating dinner. Acute coronary syndrome and pulmonary embolism were ruled out. She then developed several episodes of coffee-ground emesis while in the ED and 50cc of bright red blood was drained through a nasogastric tube. An emergency upper endoscopy revealed a feline esophagus and a linear esophageal tear. No biopsies were taken at that time due to acute bleeding. The esophageal tear was treated with epinephrine and three surgical clips. An esophagogastroduodenoscopy was performed four weeks later. Histological sections of the esophagus showed basal cell hyperplasia and intraepithelial eosinophils greater than 20/HPF. The patient was treated with a proton pump inhibitor and a swallowable topical steroid intermittently with variable response. She was referred to the allergy/immunology clinic for further evaluation three years later. She was initially restarted on a proton pump inhibitor and repeat endoscopy 8 weeks later showed possible esophageal rings consistent with EoE and 20 eosinophils/HPF. The clinical picture along with pathological findings was consistent with a diagnosis of EoE. She is currently awaiting reevaluation for additional medication treatment.

After the initial presentation of the esophageal tear, the patient started to notice symptoms of dysphagia predominantly with solid foods. At least once or twice weekly, she felt that the food was impacted in her upper chest and this sensation was relieved by inducing emesis. She also reported intermittent symptoms of heartburn and oral pruritus with strawberries and eggs. She has no history of asthma or allergic rhinitis. Her past medical history was significant for esophageal spasms, diverticulitis, hiatal hernia, hypercholesterolemia, and hyperlipidemia. Family history was significant for asthma, allergic rhinitis, and atopic dermatitis. She denied any alcohol or tobacco use. A skin prick test done as part of the allergy evaluation was negative for all foods tested and positive for trees and dust mites.

## 3. Literature Review

A PubMed based search was done combining the terms “eosinophilic esophagitis complications,” “esophageal rupture,” “Boerhaave's syndrome,” and “Mallory-Weiss tear” (Table 1). We found that esophageal rupture had been previously reported in 13 patients (11 adults and 2 children) and only 1 of them had been previously diagnosed with EoE. A history of dysphagia and food impaction was seen in the majority of these patients despite no prior diagnosis of EoE. Our results were primarily in the adult population, as Boerhaave's syndrome in children younger than 18 years of age is rare and more often is caused by trauma or is iatrogenic.

## 4. Discussion

This paper presents two adult patients with spontaneous transmural esophageal rupture as the primary manifestation of EoE. The syndrome previously known as Boerhaave's syndrome is typically seen in middle aged men with 40 to 60 years of age [5]. The clinical presentation usually involves a history of alcoholism or overindulgence in foods. The triad of vomiting, lower chest pain, and subcutaneous emphysema may also be seen [6]. Esophageal rupture is not a common primary manifestation of EoE. When it occurs as a primary manifestation, it is usually preceded by an episode of food impaction with induced vomiting. Mucosal tears and lacerations have been reported in EoE patients suggesting increased fragility of the esophageal mucosa from eosinophil infiltration and subsequent remodeling. Eosinophil granules contain cytotoxic proteins that can lead to tissue damage, and transmural eosinophilic infiltration has been shown in adult EoE patients [7]. However, despite the high frequency of mucosal tears in these patients, studies have not shown a higher frequency of esophageal perforation from dilation procedures [8].

One study by Cohen et al. showed that risk factors for endoscopic complications included a prolonged history of dysphagia, presence of esophageal stenosis, and a higher density of eosinophilic infiltration [9]. These findings all have important clinical implications as they highlight the importance of dysphagia as a presenting symptom and also the role of the eosinophil. Early treatment of inflammation with topical steroids may prevent complications such as segmental esophageal stricture, narrowed lumen of the esophagus, and esophageal rupture since topical steroids can reduce the amount of eosinophils.

Our two cases and the literature review revealed that the majority of EoE patients diagnosed only after presenting with a nontraumatic esophageal perforation had a previous history of dysphagia. Patients with a history of dysphagia should warrant an evaluation for EoE as well as patients who present with spontaneous esophageal perforation to potentially avoid other complications of untreated EoE.

## Figures and Tables

**Table 1:** Literature review of esophageal rupture in eosinophilic esophagitis.

Reference	Patient age	Previous diagnosis of EoE	Prior symptoms	Presentation	Imaging/endoscopy	Treatment
Lucendo et al. [10]	36	No	Intermittent esophageal symptoms since childhood with frequent episodes of choking; seasonal bronchial asthma and known sensitivity to mustard, peanuts, grasses, and olive pollen	Meat impaction resolved by inducing vomiting followed by intense retrosternal pain	**CT with contrast** showed extensive mediastinal and subcutaneous emphysema amongst other findings suggestive of perforation of esophagus; an **endoscopy** done 9 months later showed narrowing of middle esophagus with linear furrows and cobble stoning	Thoracotomy with closure of perforation

Lucendo et al. [10]	65	No	Several-year history of intermittent esophageal symptoms not requiring treatment	Intense abdominal pain after choking on a piece of plum which was relieved after inducing vomiting	**Endoscopy **at the time showed a deep ulcer in the distal third of the esophagus and a **CXR** showed a left pleural effusion and free air around the gastric fundus	Laparotomy with closure of perforation

Predina et al. [11]	19	No	Three-year history of dysphagia and seasonal allergies	Retching following dinner, followed by hematemesis and melena 14 hours later	**Endoscopy** revealed presence of two Mallory-Weiss tears just superior to GE junction and corrugation of esophagus	Endoscopic clipping with epinephrine injection

Quiroga et al. [12]	24	Yes	Allergy to pollen and an esophageal stricture in the middle third of the esophagus secondary to eosinophilic esophagitis	Progressive chest pain, nausea, vomiting, and fever	**Spiral CT** showed intramural circumferential dissection of thoracic esophagus and periesophageal mediastinal abscess formation	Conservative management with antibiotics and parental nutrition; corticosteroid therapy was initiated after abscess resolution was demonstrated on a CT

Robles-Medranda et al. [13]	9	No	History of asthma and intermittent solid food dysphagia	Chest pain, pyrosis, and fever after an episode of food blockage	**CXR** was normal; **CT **showed a retroesophageal perforation with periesophageal fluid collection	Conservative management with antibiotics

Riou et al. [14]	26	No	Long history of dysphagia and esophageal obstruction as a child and also had history of idiosyncratic reactions to champagne and red wine	Severe constant epigastric pain following food impaction	**CXR **confirmed air in cervical tissues and **CT** showed pneumomediastinum; **gastrografin swallow** showed free contrast in peritoneal cavity; subsequent **endoscopy** showed stenosis, circular rings, and an 8 cm long longitudinal tear on the right lateral wall of the esophagus	Subtotal esophagectomy and cervical esophagogastric anastomosis were performed

Giles et al. [15]	12	No	N/A	Sore throat, dysphagia with solids, and retrosternal pain that persisted after choking on a piece of corn	**CT **with IV contrast revealed a small contained perforation without mediastinitis or pleural effusion	Nonoperative management with broad spectrum antibiotics and total parental nutrition was used

Prasad et al. [16]	54	No	Intermittent history of solid food dysphagia, heartburn, and asthma	Presented with retrosternal pain after an episode of food impaction; he induced emesis to relieve the food impaction	**CT **demonstrates free air in the mediastinum with pleural effusions and inflammatory changes around the distal esophagus; **upper endoscopy** reveals a large tear in the distal esophagus	Conservative management with IV antibiotics and bowel rest

Spahn et al. [17]	41	No	History of multiple episodes of dysphagia	Presented with dysphagia 18 hours after ingesting acetaminophen	**Esophagoscopy **showed stricture and hemorrhage; **CT** showed mediastinal air consistent with perforation	Not mentioned

Cohen et al. [18]	56	No	History of heartburn, asthma, and seasonal allergies	Progressive nausea, vomiting, and epigastric and chest pain	**CT scan **revealed air and fluid surrounding the esophagus	Closure of the perforation

Gómez-Senent et al. [19]	35	No	N/A	Dysphagia, vomiting, and epigastric pain	**Upper endoscopy **revealed impacted bean;** CT scan **showed free liquid around esophagus and pneumomediastinum	Conservative management with antibiotics

Ligouri et al. [20]	32	No	Mild solid food dysphagia	Presented with food impaction	**Upper endoscopy **revealed mucosal disruption;** CT scan **showed circumferential dissection and mediastinal emphysema	Right thoracotomy, total esophagectomy with esophagogastroplasty, and jejunostomy

Straumann et al. [21]	28	No	Ten-year history of dysphagia	Severe vomiting and hematemesis	**Upper endoscopy **showed deep mucosal tear;** CT scan **showed pneumomediastinum	Surgery and antibiotics
